# Effects of MEA Type and Curing Temperature on the Autogenous Deformation, Mechanical Properties, and Microstructure of Cement-Based Materials

**DOI:** 10.3390/ma16165651

**Published:** 2023-08-16

**Authors:** Hua Li, Zecong Zhou, Yang Wang, Yujiang Wang, Qian Tian

**Affiliations:** 1 Jiangsu Key Laboratory of Construction Materials, College of Materials Science and Engineering, Southeast University, Nanjing 211189, China; wangyujiang@cnjsjk.cn; 2 Jiangsu Sobute New Materials Co., Ltd., Nanjing 211103, Chinatianqian@cnjsjk.cn (Q.T.); 3 State Key Laboratory of High Performance Civil Engineering Materials, Jiangsu Research Institute of Building Science, Nanjing 210008, China

**Keywords:** MgO expansive agent (MEA), curing temperature, cement-based materials, autogenous deformation, strength, microstructure

## Abstract

MgO expansive agent (MEA) has the potential to meet the shrinkage compensation demands for concrete in different types of structures due to its designable reactivity and expansion properties. This study investigated the impact of three types of MEAs with different reactivities as well as curing temperature on the autogenous deformation, mechanical properties, and the microstructure of cement-based materials. The results showed that MEA type R exhibits a faster and larger hydration degree and expansion in cement mortars than MEA type M or type S in early ages under 20 °C, while when the curing temperature increases to 40 °C and 60 °C, MEA type M and type S present with significant accelerations in the hydration degree, leading to accelerated expansion rates and significantly increased expansion values compared to MEA type R. Under 40 °C, 5% MEA type M and type S present with 2.2 times and 1.1 times higher expansion in mortars than 5% MEA type R, respectively, and 8% MEA type M and type S present with 7.1 times and 5.6 times higher expansion in mortars than 8% MEA type R, respectively. Under 60 °C, 5% MEA type M and type S present 4.0 times and 3.1 times higher expansion in mortars than 5% MEA type R, respectively, and 8% MEA type M and type S present 7.0 times and 6.6 times higher expansion in mortars than 8% MEA type R, respectively. However, the increase in porosity, especially for large pores with pore size greater than 50 nm as well as the microcracks induced by the 8% dosage of MEA type M, type S, and high curing temperature of 60 °C, result in a decrease in strength of about 30% for the cement mortars. The results indicate that MEA type R is more suitable for shrinkage compensation of cement-based materials with lower temperatures, while MEA type M and type S are more suitable for shrinkage compensation of cement-based materials with higher temperatures. Under high-temperature and low-constraint conditions, the dosage of MEA needs to be strictly controlled to prevent negative effects on the microstructure and strength of cement-based materials.

## 1. Introduction

Compensating the shrinkage of concrete through the expansion produced by expansive agents is currently one of the most commonly used approaches in engineering to mitigate shrinkage cracking of concrete structures [1]. Compared with the traditional expansive agents such as calcium sulphoaluminate-type (CSA) or CaO-type expansive agent (CEA) [2,3,4], the MgO expansive agent (MEA) has several advantages including the relatively low water requirement, chemically stable hydration product, and in particular the designable expansion properties [5,6]; thus, it has the potential to meet the shrinkage compensation demand for concrete in different types of structures, and also has been explored for the self-healing applications of cementitious materials [7,8]. However, in the previous decades, the engineering application of MEA was mostly concentrated in hydraulic concrete [9,10], and its application in other mass concrete fields such as civil engineering, transportation engineering, etc., is still in its infancy [11,12,13]. The designable expansion properties of MEA have not been fully utilized in concrete engineering.

MEA with various hydration reactivities can be prepared by changing the calcination conditions [14], and the expansion properties of MEA in cement-based materials strongly depend on the microstructure and hydration reactivity of MEA, which has been extensively studied [14,15]. In general, the higher the calcination temperature and the longer the holding time, the denser the microstructure and lower the reactivity of MEA, and at room temperature, the slower the expansion rate and the lower the early expansion value produced in the cement-based materials. Apart from its own reactivity and microstructure, the expansion produced by MEA in cement-based materials is also influenced by the internal environment of the matrix such as temperature, humidity, pH, as well as mix proportions, among which the curing temperature is one of the main factors affecting the expansion of MEA [16,17,18,19,20]. Generally, cement-based materials containing MEA cured at higher temperature exhibit more rapid expansion and reach an ultimate expansion within a shorter period. In addition to the expansion properties, the incorporation of MEA and its hydration and expansion also affect the mechanical and other properties of cement-based materials. However, there are great differences in the literature about the influence of MEA on the mechanical properties of cement-based materials [21,22,23,24,25,26], as some results presented significant improvements in compressive strength after the addition of MEA [21,22], while others indicated a reduction of compressive strength of concrete at all ages studied [26]. The different effects shown in the literature may be caused by the differences in the characteristics of the MEA used, as well as the application methods (external addition or internal addition) or application scenarios, etc.

Although the origin of the expansion force of MEA is still controversial, expansion is undeniably the consequence of the hydration of MgO in MEA to form magnesium hydroxide. Clarifying the hydration behavior of MEA is a prerequisite for understanding its macroscopic expansion characteristics, and also a key to analyzing its impact on other properties. A variety of studies have been carried out on the hydration of MEA and the influencing factors, especially the effect of temperature in the calcining process and the effect of temperature in the hydration process. However, most of the quantitative studies focused on its hydration in water [27,28], while its hydration directly in cement-based materials is still mainly in the qualitative study stage [17,29]. Considering that the hydration characteristics and product morphology of MEA in pure water cannot fully represent its behavior in cement-based materials due to differences in the microenvironment [17,30,31], it is necessary to further quantify the hydration behavior of MEA directly in cement-based materials. In addition to the hydration process, the distribution and morphology of MEA and its hydration products in cement-based materials, as well as the influence of the hydration and expansion of MEA on the microstructure of cement-based materials, are also important factors in analyzing the changes of macroscopic performance.

Based on the above research status, considering that most of MEA-added mass concrete in actual engineering is under sealed curing conditions, this work firstly studied the influence of different types of MEAs on the autogenous deformation and mechanical properties of cement-based materials under different curing temperatures and sealed conditions; then, further analysis was conducted on the hydration process and product morphology of MEA in cement-based materials, as well as the influence of the hydration and expansion of MEA on the pore structure of cement-based materials. Based on the above, the reasons for changes in macroscopic properties were analyzed, in order to provide guidance for the application of MEA in different scenarios of concrete.

## 2. Materials and Methods

### 2.1. Raw Materials

Three types of MEAs were used in this study, which are all commercial products from Jiangsu Sobute New Materials Co., Ltd. (Nanjing, China), including MEA with high (or rapid), medium, and low (or slow) reactivity, labeled as type R, type M, and type S, respectively, according to CBMF 19 [32], an association standard in China. The specific reactivity values of the three types of MEAs studied were (55 ± 5) s, (150 ± 10) s, and (220 ± 10) s, respectively, which were determined using 0.07 mol/L citric acid neutralization and defined as the time required for neutralization with the citric acid solution according to DL/T 5296-2013 [33], an industry standard in China. High reactivity value, that is high reaction time, implies low reactivity. The chemical and mineral compositions of the three types of MEAs are listed in Table 1. Other raw materials include P·I 42.5 reference cement compliant to the GB 8076 standard [34], ISO standard sand according to ISO 679 [35], and tap water.

### 2.2. Test Methods

To study the mechanical and autogenous deformation properties of cement-based materials containing MEA, cement mortars containing 0, 5%, and 8% (weight percentage of cementitious materials) MEA were prepared with a water-to-binder ratio of 0.4 and a binder-to-sand ratio of 0.5. For each mix, three mortar specimens with the size of 40 mm × 40 mm × 160 mm were prepared for the measuring of compressive strength according to ISO 679 [35] or the measuring of deformation according to JGJ/T 70 [36]. Molds for autogenous deformation measuring had a hole at the center of each end face to hold a measurement stud that was to be embedded in the specimen. The mortar specimens were all molded and tested in the laboratory maintained at a temperature of (20 ± 2) °C and a relative humidity of 60% ± 5%, which meets the requirement of the ISO 679 standard [35]. After demolding at an age of 23.5 ± 0.5 h from the addition of water to the cement, the specimens for compressive strength measurement and autogenous deformation measurement were all sealed with plastic wrap and aluminum foil with the measurement stud exposed out for autogenous deformation testing, and the initial length of the specimens for deformation testing was measured within 1 h. Then, the sealed specimens were placed in large cabinets of 20 °C, 40 °C, and 60 °C, respectively, for curing. When reaching the specified test age, the specimens were taken out from the cabinets and transferred to the standard laboratory for strength or length measurement. Here, the specimens taken out from the cabinet of 40 °C and 60 °C cooled down to room temperature before measurements occurred.

To study the hydration behavior of MEA in cement-based materials and the microstructure of cement-based materials containing MEA, cement pastes with the same water-to-binder ratio and the same dosage of MEA as the mortars were prepared, and were sealed and placed in the same cabinet as the mortar for curing. At the specified test age, cement pastes were crushed into small blocks with a size less than 5 mm and hydration was terminated with isopropanol. Partial blocks after termination of hydration and vacuum drying at 40 °C were used for MIP and SEM testing, while others were crushed to a powder able to pass through the 200-mesh square mesh sieve and used for XRD testing.

Bruker D8 ADVANCE X-ray diffractometer was used, at work conditions of Cu target, 40 kV operating voltage, 30 mA operating current, 5–70° 2θ, 0.02° step size, and 0.60 s/step. The XRD–Rietveld method was used based on the TOPAS software and cement structure databases to quantitatively analyze the phase contents of cement pastes. The fitting and refinement were step-by-step and iterative processes, to minimize the difference between experimental and simulated patterns by least squares fitting method, and to make sure the responding Rwp reached an acceptable range. Chebyshev polynomial was adopted to fit the background. The March–Dollase model was used to correct the preferred orientation effect which was found in the (001) diffraction line of Ca(OH)_2_ phase et al.

## 3. Results and Discussion

### 3.1. Autogenous Deformation and Mechanical Properties of Cement-Based Materials Containing MEA

#### 3.1.1. Autogenous Deformation Properties

**Figure 1:**
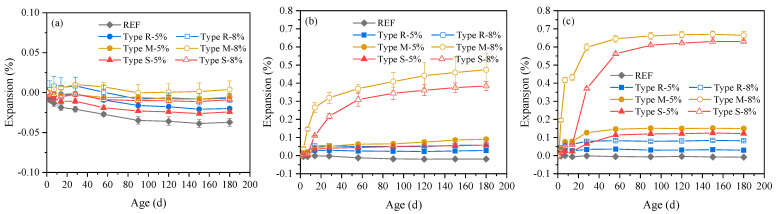
Autogenous deformation of cement mortars containing different types and dosages of MEA under the curing temperature of: (**a**) 20 °C, (**b**) 40 °C, (**c**) 60 °C.

Figure 1 shows the autogenous deformation of cement mortars containing different types and dosages of MEA under the curing temperatures of 20 °C, 40 °C, and 60 °C, respectively, in which positive values indicate expansion, and negative values indicate shrinkage. It can be seen from the comparison of Figure 1a–c that the curing temperature has a significant impact on the expansion of cement mortars containing MEA.

Specifically, with the curing temperature increases from 20 °C to 40 °C and 60 °C, the expansion rate at early ages and expansion value at later ages both significantly increased, which is different from cement-based materials containing CEA, where only the expansion rate increased while the ultimate expansion basically did not change with temperature with the range of 20 °C to 60 °C [37]. Meanwhile, the cement mortars containing different types of MEAs exhibited significantly different temperature sensitivities in expansion. More specifically, when the curing temperature increased from 20 °C to 60 °C, the 28 d and 180 d expansion of cement mortar containing 8% MEA type R increased from 0.008% and −0.007% to 0.079% and 0.083%, respectively; while the 28 d and 180 d expansion of cement mortar containing 8% MEA type M increased from 0.010% and 0.004% to 0.598% and 0.664%, respectively, and those of cement mortar containing 8% MEA type S increased from −0.002% and −0.009% to 0.369% and 0.630%, respectively.

Comparing the expansion of cement mortars containing different types of MEAs under the same curing temperatures shown in Figure 1a,b or Figure 1c, it was found that under the curing temperature of 20 °C, mortar containing MEA type R exhibited a faster and larger expansion than mortar containing MEA type M or MEA type S before 14 d; then, during the age of 14 d to 28 d, mortar containing MEA type M expanded faster, and its expansion was also the largest till the age of 180 d; while the expansion of mortar containing MEA type S was the smallest during the entire 180-day period of testing. When the curing temperature increased, the expansion rate of mortars containing MEA type M or type S accelerated and the expansion was also significantly increased compared to mortar containing MEA type R. For example, under the curing temperature of 40 °C, mortar containing MEA type M expanded the fastest and had the largest expansion at 180 d, and the expansion of mortar containing 5% MEA type M was 2.2 times larger than that of mortar containing 5% MEA type R; the expansion of mortar containing 8% MEA type M was 7.1 times larger than that of mortar containing 8% MEA type R; under the curing temperature of 60 °C, although mortar containing MEA type S presented with a lower expansion than mortar containing MEA type M at early ages, its expansion was basically close to that of mortar containing MEA type M at 180 d; the expansion of mortar containing 5% MEA type M or type S was 4.0 times or 3.1 times larger than that of mortar containing 5% MEA type R, respectively, and the expansion of mortar containing 8% MEA type M or type S was 7.0 times or 6.6 times larger than that of mortar containing 8% MEA type R, respectively. The development trend of expansion produced by MEA type R and type M in cement mortar with the increase of temperature was basically consistent with the results illustrated by Mo et al. [18], who studied the expansion of cement pastes containing MEA type R and type M cured in water with different temperatures. However, the expansion values obtained in this study were lower than those obtained by Mo et al. [18] due to the mortars specimens and sealed curing conditions studied here rather than pastes under water curing conditions.

It could be indicated from the above results that, different types of MEAs are suitable for different temperature conditions. MEA type R can produce faster and larger expansion in cement-based materials under the temperature of 20 °C, so it is more suitable for shrinkage compensation of concrete with lower temperatures, such as concrete poured in winter or thin-wall concrete. MEA type M and type S are more suitable for shrinkage compensation of concrete with higher temperatures, such as concrete poured in summer or mass concrete with temperature peaks exceeding 40 °C or even 60 °C. Additionally, it should be noted that, for MEA type S, even under the temperature of 60 °C, the expansion before 7 days is not significant, while after 7 days, the expansion increases significantly. Therefore, MEA type S is more suitable for thermal shrinkage compensation of concrete in which the temperature peak exceeds 60 °C for several days, such as mass concrete using insulation measures which has a slower temperature drop rate.

The expansion of mortar containing MEA also increases with the MEA dosage, especially for mortar containing MEA type M or MEA type S cured under the temperature of 40 °C and 60 °C, as shown in Figure 1b,c. With the MEA dosage increases from 5% to 8%, the 180 d expansion of mortar containing MEA type M increases by 4 times under 40 °C and 3.5 times under 60 °C, and the 180 d expansion of mortar containing MEA type S increases by 5.5 times under 40 °C and 4.2 times under 60 °C, while the 180 d expansion of mortar containing MEA type R only increases by 1.1 times under 40 °C and 1.8 times under 60 °C. Although an increase in MEA dosage will result in greater expansion, which is beneficial for shrinkage compensation of cement-based materials, it should be noted that when the constraint is small, excessive expansion may also cause microscopic damage, thereby affecting other properties of cement-based materials such as strength. Therefore, it is necessary to analyze the impact on strength simultaneously with deformation analysis.

#### 3.1.2. Mechanical Properties

**Figure 2:**
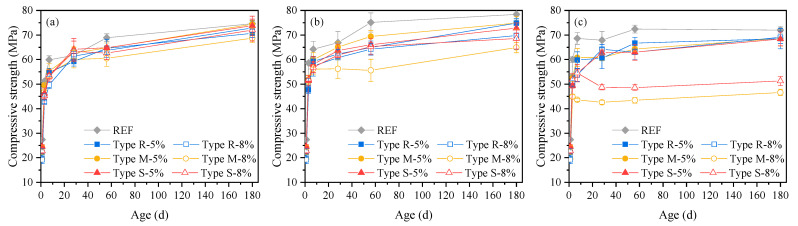
Compressive strength of cement mortars containing different types and dosages of MEAs under the curing temperatures of: (**a**) 20 °C, (**b**) 40 °C, (**c**) 60 °C.

Figure 2 shows the compressive strength of cement mortars containing different types and dosages of MEAs under the curing temperatures of 20 °C, 40 °C, and 60 °C, respectively.

It can be seen from Figure 2a that, under the curing temperature of 20 °C, cement mortars containing MEA all show lower compressive strength values than the reference mortar before 7 d; while, during the 7 d to 28 d period, cement mortars containing MEA show a more rapid strength increase than the reference mortar, and at 28 d, the compressive strengths of mortar containing 5% MEA type M and 5%, 8% MEA type S are slightly higher than that of the reference mortar, while the compressive strengths of mortar containing 5%, 8% MEA type R, and 8% MEA type M are slightly lower than that of the reference mortar. It can be inferred from the deformation shown in Figure 1a and strength shown in Figure 2a that proper expansion is beneficial for strength growth and increases the growth rate of strength, while the faster early expansion rate and higher expansion value may also lower the strength of mortar containing MEA. At 180 d, the strengths of mortars containing 5% MEA type M and 5% MEA type S are equivalent to that of the reference mortar, while the strengths of mortars containing 5% MEA type R and 8% MEA of all types are all slightly lower than that of the reference mortar, and the strength of mortar containing 8% MEA type M is the lowest, which is also consistent with the largest expansion it produces as shown in Figure 1a. It can be seen from Figure 2b,c that under the curing temperatures of 40 °C and 60 °C, the mortars containing MEA all show lower strength than the reference mortar, among which the mortar containing 8% MEA type M shows the lowest strength under 40 °C, the mortars containing 8% MEA type M and 8% MEA type S show the lowest strength under 60 °C, and the strength of the mortar containing 8% MEA type S decreases in a certain degree during 7 d to 28 d under 60 °C. It is also consistent with the development trend of expansion shown in Figure 1, that is, the mortar containing 8% MEA type M shows the largest expansion under 40 °C, the mortars containing 8% MEA type M and MEA type S show the largest expansion under 60 °C, and the mortar containing 8% MEA type S shows a larger growth in expansion during 7 d to 28 d. Additionally, it can also be seen that under the same curing temperature, the dosage increase from 5% to 8% for MEA type M and type S decreases the strength of the mortar, while the influence of the dosage increasing from 5% to 8% for MEA type R is not obvious.

**Figure 3:**
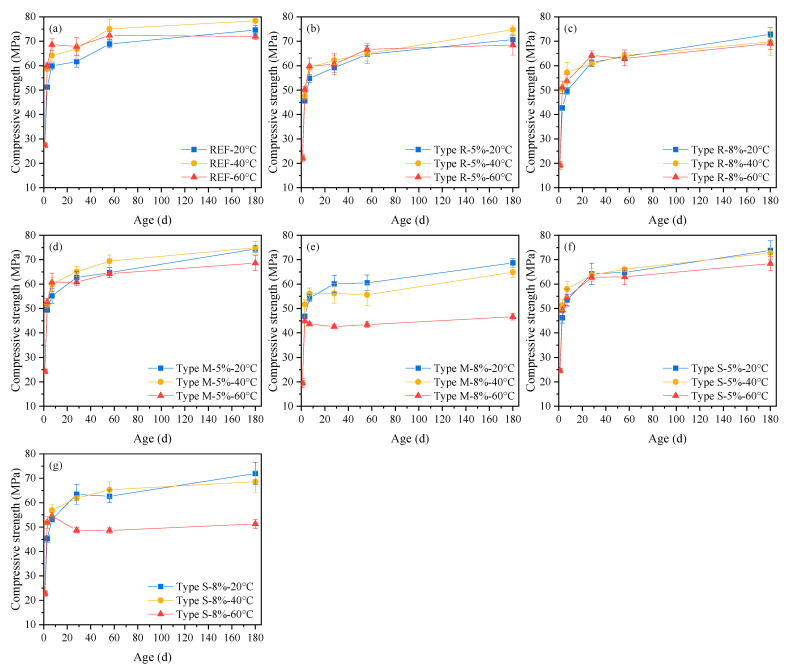
Compressive strength of cement mortars containing the same type and dosages of MEA under different curing temperatures: (**a**) REF, (**b**) MEA type R-5%, (**c**) MEA type R-8%, (**d**) MEA type M-5%, (**e**) MEA type M-8%, (**f**) MEA type S-5%, (**g**) MEA type S-8%.

Figure 3 gives the compressive strength of cement mortars containing the same type and dosage of MEA under different curing temperatures.

It is shown that, for the reference mortars, the increase in curing temperature increases the compressive strength before 28 d, while at 56 d, the strength of mortar cured under 60 °C is slight lower than that under 40 °C, and at 180 d, the strength of mortar cured under 60 °C is both lower than that under 40 °C and slight lower than that under 20 °C, which is the same as illustrated in other studies [38,39], that elevated temperatures may lead to lower final strengths; for mortars containing 5% MEA type R, type M, type S, or 8% MEA type R, the strength also increases with the increase in curing temperature before 28 d, at 28 d, and 56 d, the change in strength with temperature is not significant, and to 180 d, the strengths under 40 °C are the highest or equivalent to 20 °C, while the strengths under 60 °C are the lowest; for mortars containing 8% MEA type M or type S, though the increase in curing temperature increases the strength before 7 d, the strength of 28 d and later ages all decrease with the increase in curing temperature, and there is almost no increase in strength from 28 d to 180 d under 60 °C. Comparing the influence of MEA dosage, it is found that overall, at the MEA dosage of 5%, the increase in curing temperature creates a small impact on the strength of the mortar, while when the MEA dosage increases to 8%, a high curing temperature of 60 °C significantly exacerbates the loss of strength, especially for mortar containing MEA type M and type S. Specifically, compared to mortars under 20 °C, the 180 d strength of mortars containing 5% MEA type M or type S under 60 °C decreased by 7.8% or 7.3%, respectively; while the 180 d strength of mortars containing 8% MEA type M or type S under 60 °C decreased by 32.2% or 28.7%, respectively. Combining the test results of deformation and compressive strength, they show that the strength and expansion deformation basically demonstrate a reverse change law under high curing temperature, which indicates that, under free conditions, the increase in MEA dosage (especially when the MEA dosage is high) and high-temperature curing are accompanied by a significant loss of mechanical properties when increasing and accelerating expansion.

### 3.2. Hydration Behavior of MEA in Cement-Based Materials

**Figure 4:**
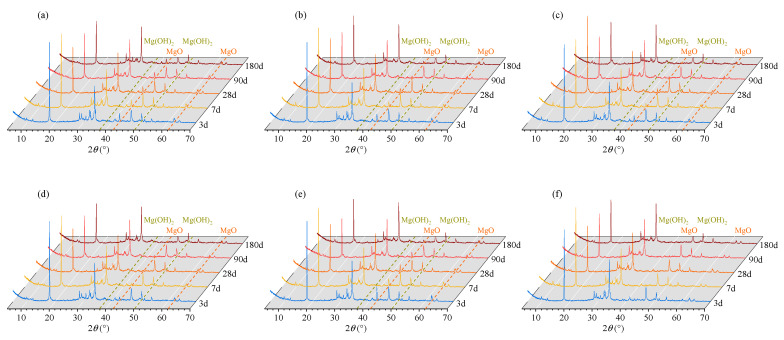
Partial XRD patterns of cement pastes containing 8% MEA of different types: (**a**) MEA type M curing under the temperature of 20 °C, (**b**) MEA type M curing under the temperature of 40 °C, (**c**) MEA type M curing under the temperature of 60 °C, (**d**) MEA type R curing under the temperature of 40 °C, (**e**) MEA type S curing under the temperature of 40 °C, (**f**) REF pastes curing under the temperature of 40 °C.

Partial XRD patterns of REF cement pastes and cement pastes containing 8% MEA of different types are shown in Figure 4.

**Figure 5:**
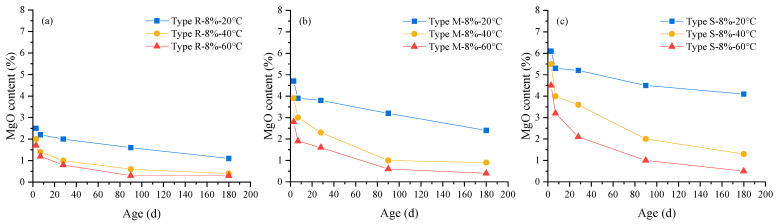
The changes of MgO content in cement pastes containing 8% MEA with age under different curing temperatures: (**a**) MEA type R, (**b**) MEA type M, (**c**) MEA type S.

**Figure 6:**
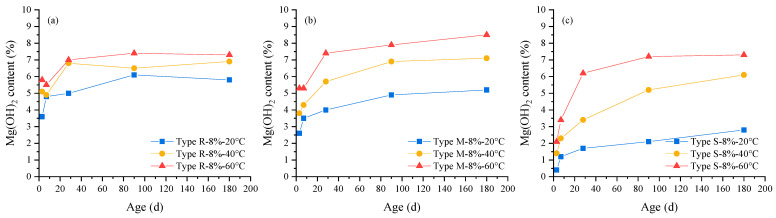
The changes of Mg(OH)_2_ content in cement pastes containing 8% MEA with age under different curing temperatures: (**a**) MEA type R, (**b**) MEA type M, (**c**) MEA type S.

The phase contents of cement pastes containing MEA at different curing ages were quantitatively analyzed by the XRD–Rietveld method based on the TOPAS software and cement structure databases. Rwp factor values of all fitting results were in the range of 9–10, illustrating the analysis results are reliable. After the fitting and refinement process being completed, computed, and the experimental spectra showing small differences, substantially no residual diffraction peaks existed, illustrating that the control files used for fitting are reasonable. The changes of MgO and Mg(OH)_2_ content with age obtained by the Rietveld method are shown in Figure 5 and Figure 6.

It can be seen from Figure 5 and Figure 6 that as the curing age increases, the MgO content gradually decreases and the Mg(OH)_2_ content gradually increases; under the same curing age, the MgO content significantly decreases and the Mg(OH)_2_ content significantly decreases with the increase in curing temperature from 20 °C to 40 °C and 60 °C. Comparing the three types of MEA, it is found that the MgO and Mg(OH)_2_ contents in the cement paste containing MEA type S present the largest variation with temperature, followed by those in the cement paste containing MEA type M, and the MgO and Mg(OH)_2_ contents in the cement paste containing MEA type R show the smallest variation with temperature.

**Figure 7:**
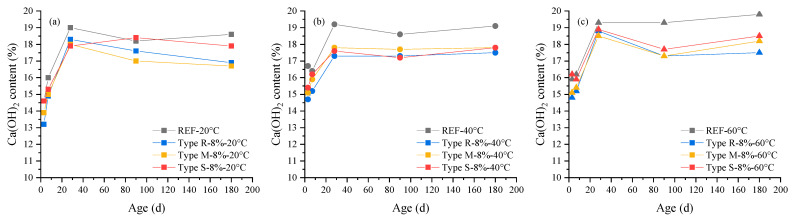
The changes of Ca(OH)_2_ content in cement pastes containing 8% MEA with age under the curing temperature of: (**a**) 20 °C, (**b**) 40 °C, (**c**) 60 °C.

The changes of Ca(OH)_2_ content with age are shown in Figure 7. It shows that the increase in curing temperature increases the Ca(OH)_2_ produced at the early ages, but has little effect on Ca(OH)_2_ content at later ages. The Ca(OH)_2_ contents in cement pastes containing MEA are all lower than those in the reference cement pastes. This is because the MEA is added to cement pastes by partially replacing the cement, resulting in a decrease in the cement content in the pastes, which in turn leads to a decrease in the Ca(OH)_2_ content generated by the hydration of the cement. Additionally, the content of ettringite generally decreases with the increase in temperature due to instability at high temperatures, but is generally lower than 2% in all pastes. The content of CaCO_3_ in all pastes does not exceed 1.5% and has no significant correlation with temperature.

**Figure 8:**
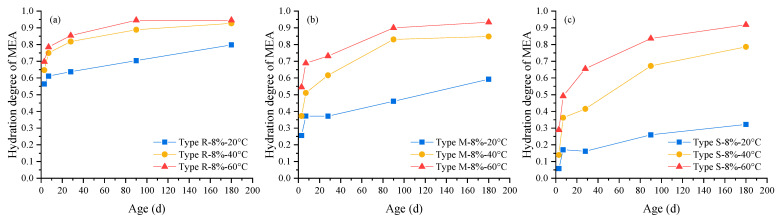
Hydration degree of MEA in cement pastes under different curing temperatures: (**a**) MEA type R, (**b**) MEA type M, (**c**) MEA type S.

Based on the MgO content shown in Figure 5, the hydration degree of three types of MEAs in cement pastes under different curing temperatures was calculated using Equation (1) with the results shown in Figure 8.

(1) $$\alpha_{\mathrm{MEA}}=1-c_{p-MEA,f-MgO}/[(1-LOI_{p-MEA})*\varphi_{MEA}*c_{0,f-MgO}]$$

where, $\alpha_{MEA}$ is the hydration degree of MEA in cement pastes, $c_{p-MEA,f-MgO}$ is the content of MgO phase in cement pastes containing MEA as shown in Figure 5, $LOI_{p-MEA}$ is the loss on ignition of cement pastes containing MEA tested according to the GB/T 176 standard [40], $\varphi_{MEA}$ is the MEA dosage (8% in this study) in cement pastes, $c_{0,f-MgO}$ is content of MgO phase in unhydrated MEA as given in Table 1.

It can be seen from Figure 8 that the hydration degrees of three types of MEAs in cement pastes all increase with the increase in curing temperature.

In comparison, the hydration degree of MEA type S increases the most with the increase in temperature, followed by MEA type M, and the hydration degree of MEA type R increases the least with the increase in temperature, indicating that the hydration behaviors of MEA type S and type M in the cement-based materials are more sensitive to temperature than that of MEA type R. Comparing the specific hydration degrees of the three types of MEAs in cement pastes under the same temperature, it was found that, under the temperature of 20 °C, the 28 d hydration degrees of MEA type R, type M, and type S were 0.64, 0.37, and 0.16, respectively, that is, MEA type R can reach a high hydration degree at early ages, while MEA type M and type S only can reach a much smaller hydration degree under 20 °C. This is why MEA type R can produce larger early-age expansion in cement mortars, while MEA type S can only produce smaller expansion in cement mortars, as shown in Figure 1a. With the temperature increase to 40 °C, the 28 d hydration degrees of MEA type R, type M, and type S reached 0.82, 0.62, and 0.42, respectively; and with the further temperature increase to 60 °C, the 28 d hydration degrees of MEA type R, type M, and type S reached 0.85, 0.73, and 0.66, respectively. The obvious increase in hydration degrees of MEA type M and type S under high temperatures led to the significant increase in expansion produced in the cement mortars as shown in Figure 1b,c. It also can be found from Figure 7 that MEA type R reached the highest hydration degree under all temperatures, while the expansion it produced in cement mortars was much smaller than MEA type M and MEA type S under high temperatures. More significantly, when the age reached 180 d, the hydration degrees of the three types of MEAs was similar under 60 °C (0.95, 0.93, and 0.92 for MEA type R, type M, and type S, respectively), while the expansion produced by MEA type M and MEA type S was 7 times and 6.6 times higher than that produced by MEA type R in cement mortars, respectively. The test results indicate that under the same hydration degree, MEAs with higher reactivity values possess greater expansion efficiencies. This provides information for the quantitative modelling and prediction of the expansion process of MEAs in cement-based materials, which will be given in further papers.

### 3.3. Microstructure of Cement-Based Materials Containing MEAs

#### 3.3.1. Pore Structures

**Figure 9:**
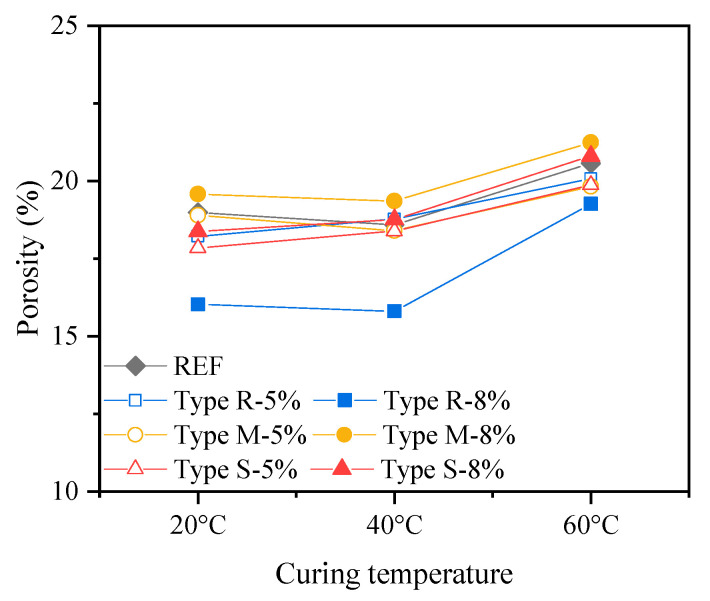
Porosity of cement pastes containing different types and dosages of MEAs at 28 d.

**Figure 10:**
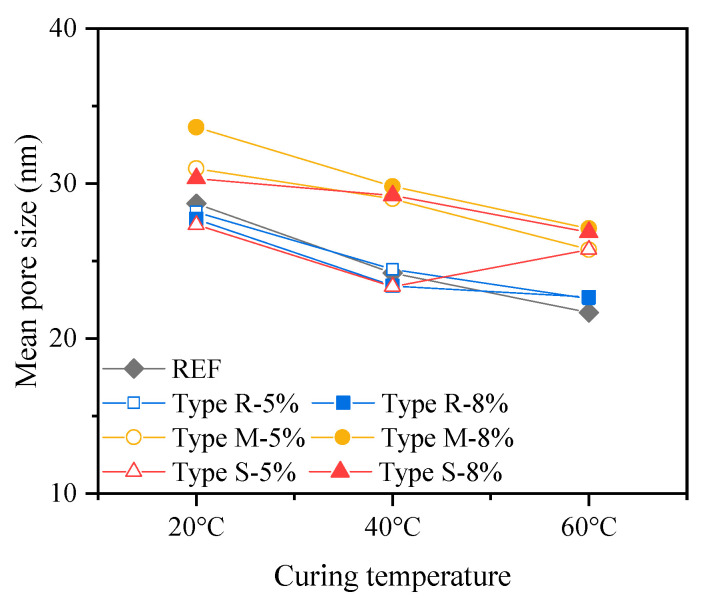
Mean pore size of cement pastes containing different types and dosages of MEAs at 28 d.

**Figure 11:**
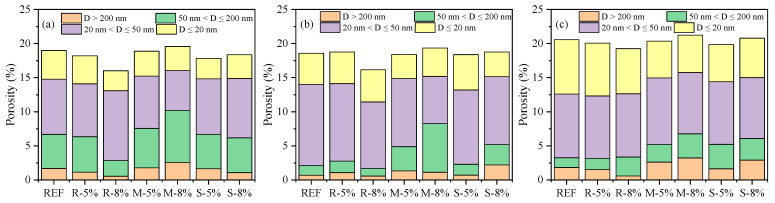
Pore size classification of cement pastes containing different types and dosages of MEAs at 28 d under the curing temperatures of: (**a**) 20 °C, (**b**) 40 °C, (**c**) 60 °C.

To investigate the impact of the incorporation and expansion of MEAs on the microstructure of cement-based materials, the porosity and pore size distribution of cement pastes containing different types and dosages of MEAs at 28 d were studied by MIP with the porosity shown in Figure 9, mean pore size shown in Figure 10, and pore size distribution shown in Figure 11.

The cumulative porosity given in Figure 9 indicates that with the curing temperature increase from 20 °C to 40 °C, the 28 d cumulative porosity of the cement pastes containing MEA slightly decreases, while when the curing temperature further increases to 60 °C, the 28 d cumulative porosity of the cement pastes containing MEA increases, even higher than those under 20 °C. Comparing the cumulative porosity of cement pastes containing different types of MEAs, it shows that the 28 d porosities of cement pastes containing 5% and 8% MEA type R both decrease compared to that of the reference cement paste, and the cement paste containing 8% MEA type R presents a greater reduction in porosity than the cement paste containing 5% MEA type R; the 28 d cumulative porosity of cement paste containing 5% MEA type M is basically equivalent to that of the reference paste, while when the dosage of MEA type M increases to 8%, the 28 d cumulative porosity of the cement paste increases compared to that of the reference paste; the 28 d porosity of cement paste containing 5% MEA type S also slight decreases compared to that of the reference cement paste, but when the dosage of MEA type S increases to 8%, the 28 d porosity increases compared to that of the reference cement paste under 60 °C.

The mean pore size presented in Figure 10 reveals that the 28 d mean pore sizes of cement pastes containing MEAs decrease slightly with the increase in curing temperature, but the magnitude order of the average pore size remains unchanged. Comparing the mean pore size of cement pastes containing different types of MEAs, it shows that the 28 d mean pore sizes of cement pastes containing 5%, 8% MEA type R, and 5% MEA type S are basically equivalent to that of the reference paste apart from an increase under 60 °C in the paste containing 5% MEA type S, while the 28 d mean pore sizes of cement pastes containing 5%, 8% MEA type M, and 8% MEA type S are larger than that of the reference paste, among which, the mean pore size of cement paste containing 8% MEA type M increases the most.

The graded porosity of different pore size ranges given in Figure 11 shows that compared to that of the reference cement pastes, the 28 d graded porosity of the paste containing 8% MEA type R significantly decreases in the pore size range of 50–200 nm and pore size larger than 200 nm under 20 °C, and slight decreases in the pore size larger than 200 nm under 40 °C and 60 °C; while the 28 d graded porosity values of the pastes containing 5% and 8% MEA type M increase in the pore size range of 50–200 nm and pore size larger than 200 nm under 20 °C, 40 °C, and 60 °C, with the increase of the paste containing 8% MEA type M more significant; the 28 d graded porosity values of pastes containing 5% and 8% MEA type S are basically the same as the reference paste in the pore size range of 50–200 nm and pore size larger than 200 nm under 20 °C, while they increase in the pore size range of 50–200 nm and pore size larger than 200 nm under 40 °C and 60 °C, especially for the paste containing 8% MEA type S.

Based on the above analysis results, it can be concluded that the addition and expansion of 5% MEA type R has little impact on the 28 d cumulative porosity, mean pore size, as well as the pore size distribution of the cement pastes. The addition and expansion of 8% MEA type R reduces the 28 d porosity of the cement pastes, especially for the pore size range larger than 50 nm. The addition and expansion of 5% MEA type M, 5%, and 8% MEA type S have little impact on the 28 d cumulative porosity of the cement pastes; however, they increase the mean pore size and porosity with pore size larger than 50 nm when the pastes were curing under high temperatures of 40 °C and 60 °C. The addition and expansion of 8% MEA type M results in an increase in the 28 d cumulative porosity, mean pore size, as well as the porosity with pore size larger than 50 nm of the cement pastes. In addition, high-temperature curing at 60 °C increases the 28 d cumulative porosity of all pastes, but the main increase is in porosity with a pore size smaller than 50 nm, while porosity with a pore size larger than 50 nm decreases.

The results of pore structure analysis can partially reflect the changes in the macroscopic strength of cement pastes containing MEAs as shown in Figure 3. Due to the significant negative impact produced by the addition and expansion of 8% MEA type M on the cumulative porosity and especially the porosity with pore size larger than 50 nm, the 28 d strength of cement mortars decreases compared to that of the reference cement mortars in all curing temperatures; similarly, due to the negative impact on pore structures produced by the addition and expansion of 8% MEA type S under the curing temperatures of 40 °C and 60 °C, the 28 d strength of the cement mortars containing 8% MEA type S also decrease under high curing temperatures. In addition to the influence of pore structure, the addition of MEA also reduces the cement content in the mortars, which leads to a reduction in the content of cementitious C-S-H gel and other products, and the inclusion of the low cementitious Mg(OH)_2_ crystals [26] in the cementitious matrix may further reduce the strength.

#### 3.3.2. SEM Micromorphology

**Figure 12:**
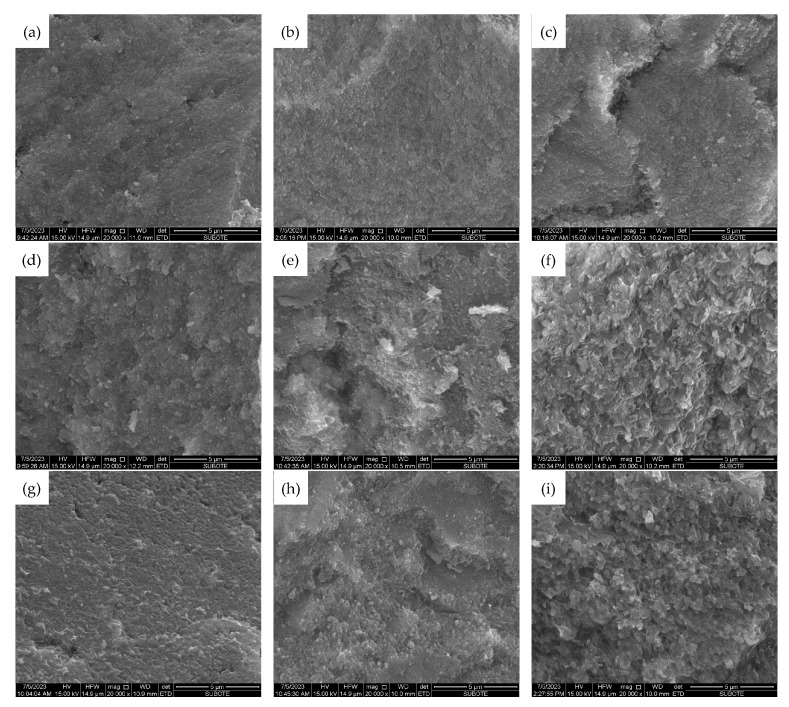
SEM micromorphology of hydration products of different types of MEAs in 180 d cement pastes cured under different temperatures: (**a**–**c**) for MEA type R cured under 20 °C, 40 °C, and 60 °C, respectively; (**d**–**f**) for MEA type M cured under 20 °C, 40 °C, and 60 °C, respectively; (**g**–**i**) for MEA type S cured under 20 °C, 40 °C, and 60 °C, respectively.

**Figure 13:**
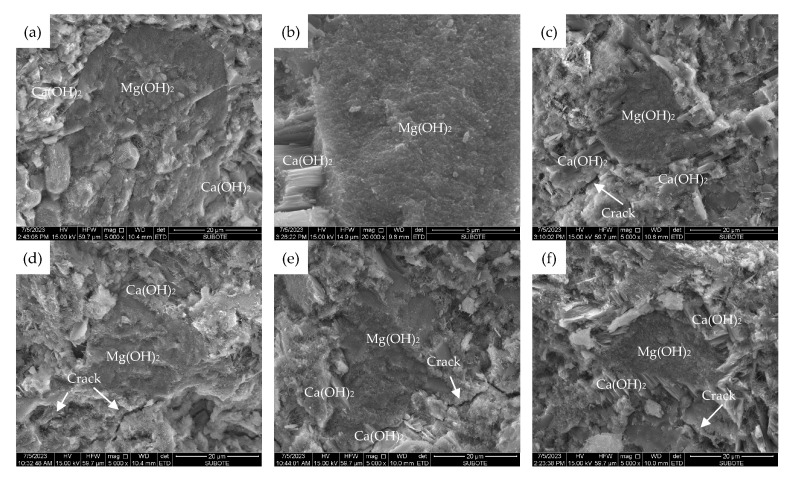
SEM micromorphology of 28 d and 180 d cement pastes containing different types of MEAs cured under different temperatures: (**a**) pastes containing MEA type R cured to 28 d under 60 °C, (**b**) pastes containing MEA type M cured to 28 d under 60 °C, (**c**) pastes containing MEA type S cured to 28 d under 60 °C, (**d**) pastes containing MEA type M cured to 180 d under 40 °C, (**e**) pastes containing MEA type S cured to 180 d under 40 °C, (**f**) pastes containing MEA type S cured to 180 d under 60 °C.

Figure 12 and Figure 13 give the SEM micromorphology of three types of MEA particles hydrated to 28 d or 180 d in cement pastes cured under 20 °C, 40 °C, and 60 °C, respectively.

It can be seen from Figure 12 that the 180 d morphology of the hydration products Mg(OH)_2_ of MEA type R in cement-based materials does not differ significantly under different curing temperatures, while the sizes of the hydration products of MEA type M and type S gradually increase with the increase in curing temperature, and the morphology of Mg(OH)_2_ changes from small thin flakes to larger flakes and plates. Meanwhile, Figure 13 indicates that there is often an enrichment of Ca(OH)_2_ around the hydration product Mg(OH)_2_ which may be due to the high concentration of hydroxyl OH^−^ in this region. These phenomena may also have an impact on the macroscopic deformation and strength of cement-based materials containing MEA. Additionally, as shown in Figure 13, several microcracks can be found near the MEA particles in the cement pastes containing MEA type M or type S cured under 40 °C or 60 °C, indicating that excessive expansion produced by MEA leads to the damage of the microstructure in the matrix, which further results in decreases in macroscopic mechanical properties.

## 4. Conclusions

Considering that MEAs with different hydration reactivities can be obtained by controlling the calcination conditions, the impact of three types of MEAs with different reactivity values as well as curing temperatures on the autogenous deformation, mechanical properties, and the microstructure of cement-based materials was investigated in this study.

It could be concluded from the results that, different types of MEAs exhibit significantly different temperature sensitivities in hydration behavior and microstructure evolution in cement-based materials, leading to different impacts on macroscopic expansion and mechanical properties. Under 20 °C, MEA type R exhibits a faster and larger hydration degree and expansion in cement mortars than MEA type M or type S in early ages, while under 40 °C and 60 °C, MEA type M and type S display significant acceleration in the hydration degree leading to accelerated expansion rates and significantly increased expansion values compared to MEA type R. Under the same hydration degree, MEAs with higher reactivity values possess greater expansion efficiency values than MEAs with lower reactivity values. The increase in porosity, especially for large pores with pore size greater than 50 nm, as well as the microcracks induced, due to the large dosage of MEA type M, type S, and high curing temperature, results in varying degrees of decrease in strength of the cement-based materials. The difference in morphology of the products Mg(OH)_2_ of different types of MEAs caused by curing temperatures may also contribute to the changes in macroscopic deformation and strength.

It is indicated from this study that MEA type R is more suitable for shrinkage compensation of cement-based materials with lower temperatures, while MEA type M and type S are more suitable for shrinkage compensation of cement-based materials with higher temperatures. Especially, MEA type S is more suitable for thermal shrinkage compensation of concrete in which the temperature peak exceeds 60 °C for several days, such as mass concrete using insulation measures which has a slower temperature drop rate. Under low-constraint conditions, the dosage of MEA needs to be strictly controlled to prevent negative effects on the microstructure and strength of cement-based materials. Further research will focus on the numerical simulation and prediction of the expansion produced by MEA produced in cement-based materials.

## Figures and Tables

**Table 1:** Chemical and mineral compositions of MEAs.

Index		Type R	Type M	Type S
Reactivity Value (s)		55 ± 5	150 ± 10	220 ± 10
Chemical compositions (XRF, mass-%)	MgO	89.16	90.40	91.38
	CaO	1.86	2.13	2.21
	SiO_2_	3.66	3.89	3.86
	Al_2_O_3_	0.76	0.71	0.63
	Fe_2_O_3_	1.00	1.07	0.95
	SO_3_	0.04	0.03	0.02
	K_2_O	0.04	0.06	0.04
	Na_2_O	0	0	0
	TiO_2_	0.02	0.01	0.02
	P_2_O_5_	0.07	0.09	0.08
	MnO	0.06	0.07	0.06
	LOI	3.32	1.51	0.70
Mineral phases (XRD, mass-%)	Periclase (MgO)	87.8	95.5	97.6
	Quartz (SiO_2_)	1.6	1.0	1.0
	Magnesite (MgCO_3_)	1.9	0.4	0.2
	Dolomite (CaMg(CO_3_)_2_)	0.9	0.5	0.3
	Calcite (CaCO_3_)	2.5	0.2	0.1
	Forsterite (Mg_2_SiO_4_)	1.3	1.8	0.8

## Data Availability

The figures with available data can be found in the attachment document.
